# Nasopharyngeal Bacterial Interactions in Children

**DOI:** 10.3201/eid1811.111904

**Published:** 2012-11

**Authors:** Qingfu Xu, Anthony Almudervar, Janet R. Casey, Michael E. Pichichero

**Affiliations:** Author affiliations: Rochester General Hospital Research Institute, Rochester, New York, USA (Q. Xu, M.E. Pichichero),; University of Rochester Medical Center, Rochester (A. Almudervar);; Legacy Pediatrics, Rochester (J.R. Casey)

**Keywords:** Streptococcus pneumoniae, Haemophilus influenzae, Moraxella catarrhalis, Staphylococcus aureus, bacteria, nasopharyngeal bacterial infections, polymicrobial colonization, bacterial interaction, acute otitis media, children

## Abstract

TOC summary: Pathogen prevalence differs during periods of health and at onset of acute otitis media.

## Medscape CME Activity

Medscape, LLC is pleased to provide online continuing medical education (CME) for this journal article, allowing clinicians the opportunity to earn CME credit. 

This activity has been planned and implemented in accordance with the Essential Areas and policies of the Accreditation Council for Continuing Medical Education through the joint sponsorship of Medscape, LLC and Emerging Infectious Diseases. Medscape, LLC is accredited by the ACCME to provide continuing medical education for physicians.

Medscape, LLC designates this Journal-based CME activity for a maximum of 1 *AMA PRA Category 1 Credit(s)*^TM^. Physicians should claim only the credit commensurate with the extent of their participation in the activity.

All other clinicians completing this activity will be issued a certificate of participation. To participate in this journal CME activity: (1) review the learning objectives and author disclosures; (2) study the education content; (3) take the post-test with a 70% minimum passing score and complete the evaluation at www.medscape.org/journal/eid; (4) view/print certificate.

**Release date: October 19, 2012; Expiration date: October 19, 2013**

## Learning Objectives

Upon completion of this activity, participants will be able to:

- Describe patterns of nasopharyngeal bacterial colonization and interaction in healthy young children, based on an observational study
- Describe the role of *Haemophilus influenzae* in acute otitis media (AOM) affecting young children, based on an observational study
- Describe the role of other bacteria in AOM affecting young children, based on an observational study

## CME Editor

**P. Lynne Stockton, VMD, MS, ELS(D), **Technical Writer/Editor, *Emerging Infectious Diseases. Disclosure: P. Lynne Stockton, VMD, MS, ELS(D), has disclosed no relevant financial relationships.*

## CME Author

**Laurie Barclay, MD, **freelance writer and reviewer, Medscape, LLC. *Disclosure: Laurie Barclay, MD, has disclosed no relevant financial relationships.*

## Authors

*Disclosures:* ***Qingfu Xu, PhD; Anthony Almudervar, PhD;*** *and* ***Michael E. Pichichero, MD,*** *have disclosed no relevant financial relationships.* ***Janet R. Casey, MD,*** *has disclosed the following relevant financial relationships: received grants for clinical research from Pfizer, Sanofi-Aventis.*
