# Supplementary material for: Nasopharyngeal Bacterial Interactions in Children
Source: Emerg Infect Dis. 2012 Nov;18(11):1738–45. doi: 10.3201/eid1811.111904 (PMC3559157; doi:10.3201/eid1811.111904)
Supplement: Related texts

# Research article: Nasopharyngeal Bacterial Interactions in Children

**Authors:** Qingfu Xu, Anthony Almudervar, Janet R. Casey, Michael E. Pichichero

**Affiliations:** Author affiliations: Rochester General Hospital Research Institute, Rochester, New York, USA (Q. Xu, M.E. Pichichero),; University of Rochester Medical Center, Rochester (A. Almudervar);; Legacy Pediatrics, Rochester (J.R. Casey)

## Abstract

Antimicrobial treatments and vaccines can alter bacterial interactions in the nasopharynx, thereby altering disease processes. To better understand these interactions, we examined colonization rates of 3 respiratory bacterial pathogens among 320 children when healthy and at onset of acute otitis media (AOM). Bacterial interactions were analyzed with a repeated measures logistic regression model. Among healthy children, *Streptococcus pneumoniae* and *Moraxella catarrhalis* were synergistically (positively) associated. Colonization with *S. pneumoniae* when healthy, but not at onset of AOM, was competitively (negatively) associated with *Staphylococcus aureus.* Among children with AOM, competitive associations were found between *Haemophilus influenzae* and *S. pneumoniae* and between *H. influenzae* and *M. catarrhalis*; rates of colonization with *H. influenzae* were higher. Bacterial interactions result in differing pathogen prevalence during periods of health and at onset of AOM. *H. influenzae* might become a more common cause of AOM among children who receive pneumococcal conjugate vaccine.

Respiratory bacterial infections, including pneumonia, acute exacerbations of bronchitis, acute sinusitis, and acute otitis media (AOM) among children and adults create major clinical concerns (*1*,*2*). The most common bacteria that cause upper respiratory tract infections are *Streptococcus pneumoniae, Haemophilus influenzae*, and *Moraxella catarrhalis* (*2*). The human nasopharynx is an ecologic reservoir of these and other bacteria. A broad variety of commensal bacteria and potential bacterial pathogens colonize the nasopharynx (*3*,*4*). Colonization of the nasopharynx is a first, and essential, step toward development of respiratory bacterial infections (*3*). Viruses can join the microbial mix as a prelude to secondary bacterial infections of the respiratory tract (*5*–*7*).

More information about microbial interactions in the nasopharynx is needed (*8*). These interactions can be altered by therapeutic (e.g., antimicrobial drug) and vaccine (e.g., pneumococcal conjugate vaccination) interventions, resulting in synergistic or competitive outcomes. Information about interactions of the major bacterial respiratory pathogens in the nasopharynx and the conditions conducive to progression to infection (e.g., concurrent viral upper respiratory infections) is limited. 

Microbial species can interact synergistically to promote persistence of colonization (positive, or synergistic, association) or they can compete (negative, or competitive, association) (*4*,*9*). Interactions between bacteria can alter the composition of a microbial community and affect incidence of disease (*4*). Several studies have reported competitive associations between colonized *S. pneumoniae* and *S. aureus* in the nasopharynx of children, raising concerns that eradication of *S. pneumoniae* from the nasopharynx by the heptavalent pneumococcal conjugate vaccine (PCV7) might lead to increased *S. aureus* colonization and subsequent infections (*10*–*13*). The introduction of the 13-valent pneumococcal conjugate vaccine will probably exacerbate this effect. Other variables that alter nasopharynx colonization patterns in children include age, gender, daycare attendance, history of having been breast-fed, environmental exposure to tobacco smoke, and otitis-prone condition (*14*,*15*).

Several recent reports have described interactions among the 3 major pathogens—*S. pneumoniae*, *H. influenzae*, and *M. catarrhalis*—in young children (*8*,*10*,*11*,*16*), but the results were contradictory (*9*). We investigated the interactions of these 3 pathogens in the nasopharynx of young children while healthy (healthy visits) and at onset of AOM (AOM visits). Our aims were to understand differences in nasopharynx colonization rates and bacterial interactions according to the child’s health status. 

## Materials and Methods

### Study Design and Participants

We analyzed data collected during June 2006–May 2011 from children enrolled in a 5-year prospective study supported by the National Institute of Deafness and Other Communication Disorders. In that study, healthy children with no previous episodes of pneumonia, sinusitis, or AOM were enrolled at 6 months of age from 5 middle-class, suburban pediatric practices in Rochester, New York, USA. Nasopharyngeal and oropharyngeal samples were obtained from healthy children at 6, 9, 12, 15, 18, and 24 months of age and examined for *S. pneumoniae*, *H. influenzae*, *M. catarrhalis*, and *S. aureus*. If symptoms compatible with an AOM infection developed, a tympanocentesis was performed, as described, to confirm the diagnosis (*17*). At the time of diagnosis, nasopharyngeal and oropharyngeal samples were obtained for bacterial pathogen cultures. All children received age-appropriate standard vaccinations, including pneumococcal conjugate vaccine (PCV7) (Prevnar; Wyeth Pharmaceuticals, Collegeville, PA, USA). 

We analyzed culture data from nasopharyngeal samples collected during 1,183 healthy visits and 334 AOM visits among 320 children 6–24 months of age. All samples included in this study were from children who had not received antimicrobial therapy for at least 3 weeks. Nasopharynx colonization at healthy versus AOM visits was compared among children at 6, 9, 12, 15, 18, and 24 months of age. This time frame includes peak incidence of AOM infection caused by *S. pneumoniae*, *H. influenzae*, and *M. catarrhalis.*

Nasopharyngeal and oropharyngeal samples were obtained for culture as described (*18*). The pathogens *S. pneumoniae*, *H. influenzae*, *M. catarrhalis* and *S. aureus* were isolated and identified according to the Manual of Clinical Microbiology (*19*).

The study was approved by the Institutional Review Board of the University of Rochester and the Rochester General Hospital. Written informed consent was obtained from parents or guardians before the children were enrolled.

### Statistical Analyses

The rates of nasopharynx **c**olonization among children of the same age at healthy and AOM visits were compared by using the Fisher exact test and GraphPad Prism software (www.graphpad.com). Bacterial interactions were analyzed by using repeated measures logistic regression models. Predicted outcomes of colonization with *S. pneumoniae*, *H. influenzae*, and *M. catarrhalis* were examined by using multivariate logistic regression. Generalized estimating equations were used to model exchangeable correlation within participants (*20*). Two logistic regression models (1 for healthy visits and 1 for AOM visits) were calculated by using R version 2.13.2 (www.r-project.org/). To examine the effects of covariates on each of the 3 pathogens, we modeled colonization of each pathogen separately by using the remaining 2 pathogens as predictors and including the interaction term (*8*). Because few *S. aureus* were isolated, we did not separately model colonization outcome for *S. aureus* (*8*). For each model, we estimated odds ratios (ORs) for the response pathogen given the presence of each predictor pathogen alone, then jointly; synergistic associations between bacteria are indicated by OR>1; competitive associations, by OR<1. The absence of both predictor pathogens was used as the reference condition. p≤0.05 was considered significant; p≤0.01, strongly significant; p≤ 0.1, weakly significant. The model was also used to estimate the OR for each pathogen relative to the risk factors of sex, age, daycare attendance, history of having been breast-fed, environmental exposure to tobacco smoke, and otitis-prone condition.

## Results

### Polymicrobial Nasopharynx Colonization 

Differences in nasopharynx colonization rates between healthy and AOM visits remained generally similar across the longitudinal samplings (Table 1). The rate of nasopharynx colonization by *S. pneumoniae* for children of all ages at healthy visits, when neither *H. influenzae* nor *M. catarrhalis* was present, was 14.2%; this rate did not differ statistically from that at AOM visits (14.4%; p = 0.93) (Table 1). In contrast, the rate of nasopharynx colonization by *H. influenzae* for children of all ages at AOM visits, when neither *S. pneumoniae* nor *M. catarrhalis* was present, was 19.5%; this rate was >4-fold higher than the rate at healthy visits (4.5%; p<0.0001) (Table 1). The rate of nasopharynx colonization by *M. catarrhalis* for children of all ages at healthy visits, when neither *H. influenzae* or *S. pneumoniae* was present, was 20.6%; this rate was 2-fold higher than that for AOM visits (10.5%; p<0.0001) (Table 1).

**Table 1:** Nasopharyngeal colonization of children of when healthy and at onset of acute otitis media, Rochester, NY, USA, June 2006–May 2010*

Group	Age, mean mo ± SD	No. visits	% Total visits											
			Single				Multiple					Overall		
			*Spn*	*Hflu*	*Mcat*		*Spn+ Hflu*	*Spn+ Mcat*	*Hflu+ Mcat*	*Spn+Hflu+ Mcat*		*Spn*	*Hflu*	*Mcat*
6 mo														
Healthy	6.4 ± 0.6	304	13.2	3.3	19.1		3.0	9.2	1.6	1.3		26.6	9.2	31.3
AOM	6.5 ± 1.2	66	22.7	19.7	9.1		10.6	13.6	4.5	13.6		60.6	48.5	40.9
9 mo														
Healthy	9.3 ± 0.4	237	12.7	3.0	21.1		2.5	10.5	1.3	2.5		28.3	9.3	35.4
AOM	9.0 ± 0.5	63	14.3	6.3	9.5		15.9	22.2	11.1	7.9		60.3	41.3	50.8
12 mo														
Healthy	12.3 ± 0.4	205	15.1	3.9	22.4		1.0	11.2	0.5	2.4		29.8	7.8	36.6
AOM	12.1 ± 1.1	90	15.6	24.4	8.9		10.0	15.6	5.6	5.6		46.7	45.6	35.6
15 mo														
Healthy	15.3 ± 0.5	170	17.1	5.3	19.4		2.9	13.5	1.8	2.4		35.9	12.4	37.1
AOM	15.3 ± 0.5	30	6.7	23.3	10.0		20.0	20.0	6.7	6.7		53.3	56.7	43.3
18 mo														
Healthy	18.4 ± 0.6	155	14.2	5.2	21.9		3.2	8.4	1.9	3.9		29.7	14.2	36.1
AOM	19.0 ± 1.2	42	4.8	28.6	16.7		9.5	16.7	7.1	9.5		40.5	54.8	50.0
24 mo														
Healthy	24.4 ± 0.5	112	14.3	9.8	20.5		1.8	15.2	7.1	7.1		38.4	25.9	50.0
AOM	24.9 ± 2.4	43	14.0	16.3	11.6		14.0	16.3	9.3	9.3		53.5	48.8	46.5
All ages														
Healthy	12.5 ± 5.6	1183	14.2	4.5	20.6		2.5	10.9	1.9	2.8		30.3	11.7	36.3
AOM	14.1 ± 7.0	334	14.4	19.5	10.5		12.6	17.1	7.2	8.7		52.7	47.9	43.4

*Spn, *Streptococcus pneumoniae*; *Hflu,* *Haemophilus influenzae*; *Mcat*, *Moraxella catarrhailis;* AOM, acute otitis media.

Polymicrobial colonization of the nasopharynx was significantly less common at healthy visits than at AOM visits. At the onset of an infection, polymicrobial colonization increased by 1.5–2.8-fold overall for children of all ages (p<0.05 for all). At healthy visits, the proportion of children with polymicrobial nasopharynx colonization was 18.1% (214/1,183); whereas at AOM visits, the proportion was 45.5% (152/334) (p<0.0001). A comparison of single-pathogen colonization and polymicrobial colonization at ages 6, 9, 12, 15, 18, and 24 months and differences between healthy visits and AOM visits are shown in the Figure.

**Figure:** Nasopharyngeal colonization of children with *Streptococcus pneumoniae, Haemophilus influenzae,* and *Moraxella catarrhalis* during routine doctor visits when healthy (triangles) and during visits for onset of acute otitis media (squares), Rochester, NY, USA, June 2006–May 2011. A) Single microbial colonization; B) polymicrobial colonization; C) overall colonization (single and polymicriobial). *p<0.05; **p<0.01, ***p<0.001, compared with healthy visits, by Fisher exact test.

The overall colonization rates for any of the 3 pathogens were 57.4% (679/1,183) at healthy visits and 89.8% (300/334) at AOM visits (p<0.0001). The culture-positive rates at healthy visits were 30.3% (358/1183) for *S. pneumoniae,* 11.7% (138/1,183) for *H. influenzae,* and 36.3% (429/1,183) for *M. catarrhalis*; whereas at AOM visits, rates were 52.7% (176/334), 47.9% (160/334), and 43.4% (145/334), respectively (p<0.0001 for all) (Table 1).

### Bacterial Interactions

The predicted outcome of nasopharynx colonization with *S. pneumoniae*, *H. influenzae*, and *M. catarrhalis* is shown in Table 2. At healthy visits, when colonization with *S. pneumoniae* was the predicted outcome, *S. pneumoniae* was synergistically associated with *M. catarrhalis* colonization (OR 1.42, p = 0.015) but not with *H. influenzae* colonization (OR 1.33, p = 0.28). When colonization with *H. influenzae* was the predicted outcome, no significant associations were found between *H. influenzae* and *S. pneumoniae* (OR 1.43, p = 0.19) or between *H. influenzae* and *M. catarrhalis* (OR 0.81, p = 0.43). When colonization with *M. catarrhalis* was the predicted outcome, *M. catarrhalis* colonization was synergistically associated with *S. pneumoniae* colonization (OR 1.51, p = 0.0059) but not with *H. influenzae* colonization (OR 0.83, p = 0.49).

**Table 2:** Predicted outcome of nasopharyngeal colonization among children when healthy and at onset of acute otitis media, Rochester, NY, USA, June 2006–May 2010*

Characteristics	Outcome, OR (95% CI)							
	*Spn*			*Hflu*			*Mcat*	
	Healthy	AOM		Healthy	AOM		Healthy	AOM
*Hflu* x *Mcat*								
Neither§	1.0	1.0		NA	NA		NA	NA
* Hflu*	1.33 (0.78–2.27)	**0.40**† **(0.22–0.71)**		NA	NA		NA	NA
* Mcat*	**1.42**‡ **(1.06–1.99)**	1.02 (0.58–1.79)		NA	NA		NA	NA
Both	3.40 (1.99–5.81)	0.79 (0.41–1.51)		NA	NA		NA	NA
*Spn* x *Mcat*								
Neither§	NA	NA		1.0	1.0		NA	NA
* Spn*	NA	NA		1.43 (0.83–2.45)	**0.41**† **(0.23–0.73)**		NA	NA
* Mcat*	NA	NA		0.81 (0.48–1.38)	**0.37**† **(0.19–0.71)**		NA	NA
Both	NA	NA		2.10 (1.30–3.40)	0.28 (0.15–0.52)		NA	NA
*Spn* x *Hflu*								
Neither§	NA	NA		NA	NA		1.0	1.0
* Spn*	NA	NA		NA	NA		**1.51**† **(1.12–2.03)**	1.02 (0.58–1.80)
* Hflu*	NA	NA		NA	NA		0.83 (0.49–1.41)	**0.35**† **(0.18–0.68)**
Both	NA	NA		NA	NA		2.17 (1.29–3.64)	0.68 (0.36–1.32**)**
*Saur*								
Absent§	1.0	1.0		1.0	1.0		1.0	1.0
Present	**0.55**‡ **(0.34–0.88)**	0.95 (0.43–2.08)		0.72 (0.33–1.54)	0.62 (0.19–2.00)		1.07 (0.69–1.66)	1.19 (0.52–2.70)
Male	0.0.84 (0.61–1.17)	0.85 (0.54–1.35)		0.83 (0.53–1.28)	0.75 (0.46–1.23)		1.07 (0.80–1.42)	**1.94**† **(1.21–3.11)**
Daycare	**1.87**† **(1.34–2.59)**	1.29 (0.81–2.05)		**1.71**‡ **(1.1–2.67)**	**2.06**† **(1.26–3.38)**		1.28 (0.94–1.73)	0.91 (0.58–1.45)
Breast-fed	1.29 (0.92–1.83)	1.07 (0.65–1.77)		0.75 (0.48–1.16)	0.81 (0.48–1.38)		1.00 (0.74–1.35)	1.02 (0.63–1.63)
Smoke	0.77 (0.48–1.22)	0.65 (0.31–1.34)		0.74 (0.38—1.43)	0.61 (0.29–1.30)		0.80 (0.53–1.20)	0.98 (0.41–2.30)
Otitis prone	**2.34**‡ **(1.19–4.59)**	0.98 (0.63–1.53)		**3.10**† **(1.51–6.36)**	1.42 (0.89–2.27)		1.16 (0.51–2.66)	0.98 (0.63–1.52)

*Boldface indicates significance. OR, odds ratio; *Spn*, *Streptococcus pneumoniae*; *Hflu,* *Haemophilus influenzae*; *Mcat*, *Moraxella catarrhailis*; AOM, acute otitis media; NA, not applicable; *Saur*, *Staphylococcus aureus*. Bacterial interactions were analyzed by using repeated measures logistic regression models. †p≤0.01. ‡p≤0.05. §Reference.

At onset of AOM infection, the predictions differed greatly. When colonization with *S. pneumoniae* was the predicted outcome, *S. pneumoniae* colonization was competitively associated with *H. influenzae* colonization (OR 0.40, p = 0.0014) but not with *M. catarrhalis* colonization (OR 1.02, p = 0.94). When colonization with *H. influenzae* was the predicted outcome, *H. influenzae* colonization was competitively associated with *S. pneumoniae* colonization (OR 0.41, p = 0.0021) and *M. catarrhalis* colonization (OR 0.37, p = 0.0022). When colonization with *M. catarrhalis* was the predicted outcome, *M. catarrhalis* colonization was competitively associated with *H. influenzae* colonization (OR 0.35, p = 0.0015) but not with *S. pneumoniae* colonization (OR 1.02, p = 0.95).

The higher prevalence of a potential otopathogen in the nasopharynx need not imply greater association. For example, the marginal rates for the pathogens in the healthy group (all ages) were 0.303, 0.117, and 0.363, the product of which is 0.0129 (Table 1). The actual rate for the occurrence of all 3 is 0.028, indicating an increase in association compared with chance. In contrast, the corresponding rates for the AOM group are 0.527, 0.479, 0.434, the product of which is 0.1096, compared with an actual rate of occurrence of 0.087 for all 3, indicating a decrease in association compared with chance. These totals are therefore compatible with the ORs reported in Table 2.

### Effects of Other Risk Factors on Bacterial Colonization 

Analysis of the effects of *S. aureus* colonization and host factors on nasopharynx colonization by *S. pneumoniae*, *H. influenzae*, and *M. catarrhalis* in children when healthy and at onset of AOM indicated that nasopharyngeal cultures were positive for *S. aureus* at 7.7% (91/1,183) of healthy visits and 5.7% (19/334) of AOM visits (p = 0.23). *S. aureus* was competitively associated with *S. pneumoniae* at healthy visits (OR 0.55, p = 0.011) but not at AOM visits (OR 0.95, p = 0.89). No significant associations were identified between *S. aureus* and *H. influenzae* or between *S. aureus* and *M. catarrhalis* at either type of visit (Table 2). At the healthy visits, daycare attendance was significantly positively associated with *S. pneumoniae* (OR 1.87, p = 0.0001) and *H. influenzae* colonization (OR 1.71, p = 0.015) but not with *M. catarrhalis* colonization (OR 1.28, p = 0.11). At onset of AOM, daycare attendance was significantly positively associated with *H. influenzae* colonization (OR 2.06, p = 0.0032) but not with *S. pneumoniae* (OR 1.29, p = 0.28) or *M. catarrhalis* colonization (OR 0.91, p = 0.70). The otitis-prone condition (defined as 3 episodes of AOM infection within 6 months or 4 AOM infections within 12 months) was significantly positively associated with *S. pneumoniae* (OR 2.34, p = 0.016) and *H. influenzae* colonization (OR 3.1, p = 0.0017) at healthy visits but not at AOM visits. We found no significant association between smoking exposures and colonization or between a history of breastfeeding and colonization at either type of visit (Table 2). Being male was positively associated with *M. catarrhalis* colonization (OR 1.94, p = 0.005) at AOM visits but not at healthy visits.

## Discussion

We found that patterns of nasopharynx colonization associations in children differed at onset of AOM and when healthy. *H. influenzae* colonization was competitively associated with *S. pneumoniae* and *M. catarrhalis* colonization at AOM visits but not at healthy visits; the rates of nasopharynx colonization by *H. influenzae* were 4-fold higher during AOM visits than during healthy visits.

Our findings suggest that during the PCV era, *H. influenzae* might increase as a bacterial pathogen of AOM. Our data among young children show that nasopharynx colonization studies of healthy children might not reflect the polymicrobial mix at the time of onset of AOM; the nasopharyngeal environment during onset of AOM favors *H. influenzae* colonization. It is the mix of bacteria at time of infection that determines which organisms are most likely to cause infection (*12*). We have previously shown that among children, the changes in nasopharynx colonization by *S. pneumoniae* and *H. influenzae* caused by PCV7 resulted in a remarkable proportionate decrease in AOM infection caused by *S. pneumoniae* and a proportionate increase in AOM infection caused by *H. influenzae* (*17*,*21*,*22*). At onset of AOM, when *H. influenzae* co-colonizes with *S. pneumoniae* or *M. catarrhalis, H. influenzae* predominates over these 2 bacteria to cause AOM (*18*). The elimination of *S. pneumoniae* strains expressing PCV7 serotypes has resulted in the remaining *S. pneumoniae* strains, except serotype 19A, competing less effectively with *H. influenzae* in the nasopharynx (*18*). Therefore, with a further reduction in nasopharynx colonization by *S. pneumoniae,* including strains expressing serotype 19A (as will probably result from the recent introduction of PCV13 in some countries), our data suggest that *H. influenzae* might fill the nasopharyngeal niche at the onset of AOM, and consequently, *H. influenzae* might become a more prominent cause of AOM.

*S. aureus* also can cause respiratory infections; several reports have suggested that *S. aureus* might be replacing *S. pneumoniae* as a dominant nasopharynx colonizer as a consequence of the introduction of PCV. Concern has been expressed that *S. aureus* might emerge as a more prominent respiratory pathogen (*10*–*13*,*23*). However, our results do not support that *S. aureus* has emerged as a frequent pathogen of AOM after introduction PCV7.

Knowledge regarding interactions among *S. pneumoniae*, *H. influenzae*, and *M. catarrhalis* is limited, appears contradictory, and is confined to studies among children. Zemlickova et al. found no significant association between *S. pneumoniae* and *H. influenzae* or between *S. pneumoniae* and *M. catarrhalis* in the nasopharynx of 425 healthy children, 3–6 years of age, in the Czech Republic (*11*). Madhi et al. found synergistic associations between *S. pneumoniae* and *H. influenzae* in PCV9-vaccinated healthy children, 5 years of age, in South Africa (*10*). Jacoby et al. found synergistic associations between nasopharynx colonization by *S. pneumoniae*, *H. influenzae*, and *M. catarrhalis* in healthy children, 1–24 months of age, but no significant association between *S. pneumoniae* and *S. aureus* or between *H. influenzae* and *S. aureus* (*16*). At onset of respiratory viral infections and in association with AOM infection, Pettigrew et al. (*8*) found competitive associations between colonization by *S. pneumoniae* and *H. influenzae, H. influenzae* and *M. catarrhalis, S. pneumoniae* and *S. aureus,* and *H. influenzae* and *S. aureus* in children 6–36 months of age. Thus, our results comparing nasopharynx colonization patterns during times of health with patterns at onset of AOM help explain the prior contradictory results.

Although we found a synergistic association between *S. pneumoniae and M. catarrhalis* at healthy visits and a significant increase of polymicrobial colonization at AOM visits, the tendency toward polymicrobial colonization did not result in synergistic associations at AOM visits. On the contrary, competitive associations between *H. influenzae* and *S. pneumoniae and* between *H. influenzae* and *M. catarrhalis* were found at AOM visits. The increases in colonization of individual bacterial pathogens during AOM might randomly result in an increase of polymicrobial colonization and might not result from synergistic associations among the potential pathogens. Further study on the mechanism is needed.

The mechanisms to explain competitive and synergistic interactions among *S. pneumoniae, H. influenzae, M. catarrhalis*, and *S. aureus* have been explored. In a mouse model, Lysenkoet al. found that when *H. influenzae* colonized with *S. pneumoniae* in the nasopharynx, *S. pneumoniae* was rapidly cleared (*24*). The competitive interaction was dependent on cellular components of *H. influenzae* activating the host innate immune response involving complement and neutrophils; the end result was the killing of *S. pneumoniae* (*24*). The mouse model results are consistent with our observations among children but differ from results observed during in vitro experiments that predict that *S. pneumoniae* should inhibit the growth of *H. influenzae* (*25*,*26*). A competitive association between *S. pneumoniae* and *S. aureus* might be mediated by *S. pneumoniae* production of hydrogen peroxide (*25*,*27*). Our results and those of others suggest that this mechanism might be active during times of health but not, on the basis of our observation, at onset of AOM (*10*,*11*,*13*,*28*).

We did evaluate the effects of age, sex, daycare attendance, breast-feeding history, exposure to tobacco smoke, and an immunologically driven increased susceptibility to AOM (*15*,*29*) in our study population. The results differed at healthy versus AOM visits except for the effect of daycare attendance on *H. influenzae* colonization; daycare attendance had a positive association with *H. influenzae* colonization at both healthy and AOM visits. Others have shown daycare attendance to be consistently associated with increased colonization by *S. pneumoniae, H. influenzae,* and *M. catarrhalis* in healthy children and in children with viral upper respiratory infections (*8*). Our previous studies have shown that children who are prone to otitis have much weaker immune responses to *S. pneumoniae* surface antigens than those who are not prone (*15*,*30*). We therefore assessed the otitis-prone condition as a predictor in the models in this study. We found that the otitis-prone condition was positively associated with *S. pneumoniae* and *H. influenzae* colonization at healthy visits but not at AOM visits.

The rates of nasopharynx colonization with *S. pneumoniae, H. influenzae*, and *M. catarrhalis* and the mix at onset of AOM that we observed occurred mostly in the context of a concurrent viral upper respiratory tract infection. A limitation of our study is that we did not evaluate bacterial–viral interactions. Revai et al. (*31*) and Chonmaitree et al. (*32*) found that among young children, viral upper respiratory tract infections preceded >90% of AOM infections. At onset of AOM, 93% of the children in our study had clinical signs of a viral upper respiratory tract infection; the predominant viruses detected were influenza, parainfluenza, respiratory syncytial, and adenovirus (A. Chang, unpub. data). The culture and PCR methods used in that study did not identify rhinovirus, metapneumovirus, or bocavirus. Previous studies have shown that viral upper respiratory tract infections affect bacterial nasopharynx colonization (*5*,*7*). Respiratory viruses up-regulate epithelial cell receptors for some species of respiratory bacteria (*7*); they cause inflammation and down-regulate innate and adaptive host defenses (*7*,*33*). Different viruses have varying effects on different bacteria (*34*–*36*). The comparison of healthy visits with AOM visits suggests that viral infections alter the host nasopharynx environment by facilitating a shift in the polymicrobial mix and enhancing polymicrobial colonization.

We did not study the additional effect of antimicrobial drugs on nasopharynx colonization. Such treatment modifies nasopharynx colonization patterns. Pettigrew et al. (*8*) showed that antimicrobial drug therapy was associated with a lower prevalence of colonization with *S. pneumoniae* and *M. catarrhalis* but not with *H. influenzae*. Varon et al. (*37*) showed that colonization by *S. pneumoniae, H. influenzae*, and *M. catarrhalis* decreased after antimicrobial drug thereapy; the reduction in colonization was less for *H. influenzae* than for *S. pneumoniae* or *M. catarrhalis*. Current national guidelines endorse the use of amoxicillin for first-line treatment of AOM, sinusitis, and community-acquired pneumonia in children (*38*–*40*). Amoxicillin is ineffective for eradicating β-lactamase–producing *H. influenzae* and *M. catarrhalis*. We have previously shown that ≈65% of *H. influenzae* and 100% of *M. catarrhalis* colonizing the nasopharynx of children in our study population elaborate β-lactamase (*17*,*21*,*22*). Therefore, empiric treatment with amoxicillin would probably increase *H. influenzae* and *M. catarrhalis* nasopharynx colonization in children.

Our results led us to 2 conclusions. First, nasopharyngeal bacterial interactions among *S. pneumoniae*, *H. influenzae*, and *M. catarrhalis* differ during health and at onset of AOM in young children. Second, at the onset of AOM, the nasopharynx environment among children vaccinated with PCV7 is favorable for *H. influenzae* colonization. Consequently, our results predict that *H. influenzae* might be become a more prominent bacterial pathogen of AOM in the era of PCV. Further studies of virus–bacterium–host interactions in the nasopharynx and additional studies of the mechanisms driving the observed shifts in bacterial species and polymicrobial makeup are needed.

## References

[1] Brunton S. Treating community-acquired bacterial respiratory tract infections: update on etiology, diagnosis, and antimicrobial therapy. J Fam Pract. 2005;54:357–64.15833229

[2] Cappelletty D. Microbiology of bacterial respiratory infections. Pediatr Infect Dis J. 1998;17(Suppl):S55–61. 10.1097/00006454-199808001-000029727651

[3] Leiberman A, Dagan R, Leibovitz E, Yagupsky P, Fliss DM. The bacteriology of the nasopharynx in childhood. Int J Pediatr Otorhinolaryngol. 1999;49(Suppl 1):S151–3. 10.1016/S0165-5876(99)00151-210577795

[4] Laufer AS, Metlay JP, Gent JF, Fennie KP, Kong Y, Pettigrew MM. Microbial communities of the upper respiratory tract and otitis media in children. MBio. 2011;2:e00245–10. 10.1128/mBio.00245-1021285435PMC3031303

[5] Heikkinen T, Chonmaitree T. Importance of respiratory viruses in acute otitis media. Clin Microbiol Rev. 2003;16:230–41. 10.1128/CMR.16.2.230-241.200312692096PMC153141

[6] Wald ER, Guerra N, Byers C. Upper respiratory tract infections in young children: duration of and frequency of complications. Pediatrics. 1991;87:129–33.1987522

[7] Bakaletz LO. Immunopathogenesis of polymicrobial otitis media. J Leukoc Biol. 2010;87:213–22. 10.1189/jlb.070951819843575PMC2812561

[8] Pettigrew MM, Gent JF, Revai K, Patel JA, Chonmaitree T. Microbial interactions during upper respiratory tract infections. Emerg Infect Dis. 2008;14:1584–91. 10.3201/eid1410.08011918826823PMC2609881

[9] Murphy TF, Bakaletz LO, Smeesters PR. Microbial interactions in the respiratory tract. Pediatr Infect Dis J. 2009;28(Suppl):S121–6. 10.1097/INF.0b013e3181b6d7ec19918134

[10] Madhi SA, Adrian P, Kuwanda L, Cutland C, Albrich WC, Klugman KP. Long-term effect of pneumococcal conjugate vaccine on nasopharyngeal colonization by *Streptococcus pneumoniae—*and associated interactions with *Staphylococcus aureus* and *Haemophilus influenzae* colonization—in HIV-infected and HIV-uninfected children. J Infect Dis. 2007;196:1662–6. 10.1086/52216418008250

[11] Zemlicková H, Urbásková P, Adámková V, Motlová J, Lebedová V, Procházka B. Characteristics of *Streptococcus pneumoniae*, *Haemophilus influenzae*, *Moraxella catarrhalis* and *Staphylococcus aureus* isolated from the nasopharynx of healthy children attending day-care centres in the Czech Republic. Epidemiol Infect. 2006;134:1179–87. 10.1017/S095026880600615716684402PMC2870503

[12] Bogaert D, van Belkum A, Sluijter M, Luijendijk A, de Groot R, Rumke HC, Colonisation by *Streptococcus pneumoniae* and *Staphylococcus aureus* in healthy children. Lancet. 2004;363:1871–2. 10.1016/S0140-6736(04)16357-515183627

[13] Regev-Yochay G, Dagan R, Raz M, Carmeli Y, Shainberg B, Derazne E, Association between carriage of *Streptococcus pneumoniae* and *Staphylococcus aureus* in children. JAMA. 2004;292:716–20. 10.1001/jama.292.6.71615304469

[14] Stenström C, Ingvarsson L. Otitis-prone children and controls: a study of possible predisposing factors. 2. Physical findings, frequency of illness, allergy, day care and parental smoking. Acta Otolaryngol. 1997;117:696–703. 10.3109/000164897091134629349865

[15] Sharma SK, Casey JR, Pichichero ME. Reduced memory CD4+ T-cell generation in the circulation of young children may contribute to the otitis-prone condition. J Infect Dis. 2011;204:645–53. 10.1093/infdis/jir34021791667PMC3144174

[16] Jacoby P, Watson K, Bowman J, Taylor A, Riley TV, Smith DW, Modelling the co-occurrence of *Streptococcus pneumoniae* with other bacterial and viral pathogens in the upper respiratory tract. Vaccine. 2007;25:2458–64. 10.1016/j.vaccine.2006.09.02017030494PMC7173051

[17] Casey JR, Adlowitz DG, Pichichero ME. New patterns in the otopathogens causing acute otitis media six to eight years after introduction of pneumococcal conjugate vaccine. Pediatr Infect Dis J. 2010;29:304–9.1993544510.1097/INF.0b013e3181c1bc48PMC3959886

[18] Xu Q, Casey JR, Chang A, Pichichero M. When co-colonizing the nasopharynx *Haemophilus influenzae* predominates over *Streptococcus pneumoniae* except serotype 19A strains to cause acute otitis media. Pediatr Infect Dis J. 2012;31:638–40. 10.1097/INF.0b013e31824ba6f7PMC335650622301480

[19] Murray PR, Pfaller MA, Jorgensen JH, Yolken RH, editors. Manual of clinical microbiology. 8th ed; 2003. p. 412–29, 636–48, 770–802, 390–411.

[20] Prentice RL, Zhao LP. Estimating equations for parameters in means and covariances of multivariate discrete and continuous responses. Biometrics. 1991;47:825–39. 10.2307/25326421742441

[21] Pichichero ME, Casey JR. Emergence of a multiresistant serotype 19A pneumococcal strain not included in the 7-valent conjugate vaccine as an otopathogen in children. JAMA. 2007;298:1772–8. 10.1001/jama.298.15.177217940232

[22] Casey JR, Pichichero ME. Changes in frequency and pathogens causing acute otitis media in 1995–2003. Pediatr Infect Dis J. 2004;23:824–8. 10.1097/01.inf.0000136871.51792.1915361720

[23] Veenhoven R, Bogaert D, Uiterwaal C, Brouwer C, Kiezebrink H, Bruin J, Effect of conjugate pneumococcal vaccine followed by polysaccharide pneumococcal vaccine on recurrent acute otitis media: a randomised study. Lancet. 2003;361:2189–95. 10.1016/S0140-6736(03)13772-512842372

[24] Lysenko ES, Ratner AJ, Nelson AL, Weiser JN. The role of innate immune responses in the outcome of interspecies competition for colonization of mucosal surfaces. PLoS Pathog. 2005;1:e1. 10.1371/journal.ppat.001000116201010PMC1238736

[25] Pericone CD, Overweg K, Hermans PW, Weiser JN. Inhibitory and bactericidal effects of hydrogen peroxide production by *Streptococcus pneumoniae* on other inhabitants of the upper respiratory tract. Infect Immun. 2000;68:3990–7. 10.1128/IAI.68.7.3990-3997.200010858213PMC101678

[26] Shakhnovich EA, King SJ, Weiser JN. Neuraminidase expressed by *Streptococcus pneumoniae* desialylates the lipopolysaccharide of *Neisseria meningitidis* and *Haemophilus influenzae*: a paradigm for interbacterial competition among pathogens of the human respiratory tract. Infect Immun. 2002;70:7161–4. 10.1128/IAI.70.12.7161-7164.200212438402PMC133026

[27] Regev-Yochay G, Trzcinski K, Thompson CM, Malley R, Lipsitch M. Interference between *Streptococcus pneumoniae* and *Staphylococcus aureus*: in vitro hydrogen peroxide–mediated killing by *Streptococcus pneumoniae*. J Bacteriol. 2006;188:4996–5001. 10.1128/JB.00317-0616788209PMC1482988

[28] Regev-Yochay G, Raz M, Dagan R, Porat N, Shainberg B, Pinco E, Nasopharyngeal carriage of *Streptococcus pneumoniae* by adults and children in community and family settings. Clin Infect Dis. 2004;38:632–9. 10.1086/38154714986245

[29] Sharma SK, Casey JR, Pichichero ME. Reduced serum IgG responses to pneumococcal antigens in otitis prone children may be due to poor memory B-cell generation. J Infect Dis. 2011;204:645–53..2238367510.1093/infdis/jis179PMC3308907

[30] Kaur R, Casey JR, Pichichero ME. Serum antibody response to five *Streptococcus pneumoniae* proteins during acute otitis media in otitis-prone and non-otitis-prone children. Pediatr Infect Dis J. 2011;30:645–50. 10.1097/INF.0b013e31821c2d8b21487325PMC3296524

[31] Revai K, Mamidi D, Chonmaitree T. Association of nasopharyngeal bacterial colonization during upper respiratory tract infection and the development of acute otitis media. Clin Infect Dis. 2008;46:e34–7. 10.1086/52585618205533PMC2707183

[32] Chonmaitree T, Revai K, Grady JJ, Clos A, Patel JA, Nair S, Viral upper respiratory tract infection and otitis media complication in young children. Clin Infect Dis. 2008;46:815–23. 10.1086/52868518279042PMC2744371

[33] Ratner AJ, Lysenko ES, Paul MN, Weiser JN. Synergistic proinflammatory responses induced by polymicrobial colonization of epithelial surfaces. Proc Natl Acad Sci U S A. 2005;102:3429–34. 10.1073/pnas.050059910215728393PMC552945

[34] Tong HH, Fisher LM, Kosunick GM, DeMaria TF. Effect of adenovirus type 1 and influenza A virus on *Streptococcus pneumoniae* nasopharyngeal colonization and otitis media in the chinchilla. Ann Otol Rhinol Laryngol. 2000;109:1021–7.1108999210.1177/000348940010901106

[35] Suzuki K, Bakaletz LO. Synergistic effect of adenovirus type 1 and nontypeable *Haemophilus influenzae* in a chinchilla model of experimental otitis media. Infect Immun. 1994;62:1710–8.816893210.1128/iai.62.5.1710-1718.1994PMC186390

[36] Bakaletz LO, Murwin DM, Billy JM. Adenovirus serotype 1 does not act synergistically with *Moraxella (Branhamella) catarrhalis* to induce otitis media in the chinchilla. Infect Immun. 1995;63:4188–90.755834110.1128/iai.63.10.4188-4190.1995PMC173592

[37] Varon E, Levy C, De La Rocque F, Boucherat M, Deforche D, Podglajen I, Impact of antimicrobial therapy on nasopharyngeal carriage of *Streptococcus pneumoniae*, *Haemophilus influenzae*, and *Branhamella catarrhalis* in children with respiratory tract infections. Clin Infect Dis. 2000;31:477–81. 10.1086/31398110987708

[38] Cincinnati Children’s Hospital Medical Center. Evidence-based care guideline for community acquired pneumonia in children 60 days through 17 years of age. Rockville (MD): Agency for Healthcare Research and Quality, National Guideline Clearinghouse; 2001 (revised 2006).

[39] Cincinnati Children’s Hospital Medical Center. Evidence-based care guideline for management of acute bacterial sinusitis in children 1 to 18 years of age. Rockville (MD): Agency for Healthcare Research and Quality, National Guideline Clearinghouse; 2000 (revised 2006).

[40] University of Michigan Health System. Otitis media. Rockville (MD): Agency for Healthcare Research and Quality, National Guideline Clearinghouse; 2002 (revised 2007).

# Research article: Nasopharyngeal Bacterial Interactions in Children

**Authors:** Qingfu Xu, Anthony Almudervar, Janet R. Casey, Michael E. Pichichero

**Affiliations:** Author affiliations: Rochester General Hospital Research Institute, Rochester, New York, USA (Q. Xu, M.E. Pichichero),; University of Rochester Medical Center, Rochester (A. Almudervar);; Legacy Pediatrics, Rochester (J.R. Casey)

## Medscape CME Quiz

## Earning CME Credit

To obtain credit, you should first read the journal article. After reading the article, you should be able to answer the following, related, multiple-choice questions. To complete the questions (with a minimum 70% passing score) and earn continuing medical education (CME) credit, please go to www.medscape.org/journal/eid. Credit cannot be obtained for tests completed on paper, although you may use the worksheet below to keep a record of your answers. You must be a registered user on Medscape.org. If you are not registered on Medscape.org, please click on the New Users: Free Registration link on the left hand side of the website to register. Only one answer is correct for each question. Once you successfully answer all post-test questions you will be able to view and/or print your certificate. For questions regarding the content of this activity, contact the accredited provider, CME@medscape.net. For technical assistance, contact CME@webmd.net. American Medical Association’s Physician’s Recognition Award (AMA PRA) credits are accepted in the US as evidence of participation in CME activities. For further information on this award, please refer to http://www.ama-assn.org/ama/pub/category/2922.html. The AMA has determined that physicians not licensed in the US who participate in this CME activity are eligible for *AMA PRA Category 1 Credits*™. Through agreements that the AMA has made with agencies in some countries, AMA PRA credit may be acceptable as evidence of participation in CME activities. If you are not licensed in the US, please complete the questions online, print the certificate and present it to your national medical association for review.

## Article Title:  Nasopharyngeal Bacterial Interactions in Children

## CME Questions

**1. Based on the observational study by Dr. Xu and colleagues, which of the following statements about patterns of nasopharyngeal bacterial colonization and interaction in healthy young children is most likely correct?**

A. *Streptococcus pneumoniae* is positively associated with *Staphylococcus aureus*

B. *S. pneumoniae* is positively associated with *Moraxella catarrhalis*

C. *M. catarrhalis* is positively associated with *S. aureus*

D. Patterns of nasopharyngeal bacterial colonization and interaction in health are likely to predict those at onset of acute otitis media (AOM)

**2. Your patient is a 1-year-old, PCV7-vaccinated male with AOM. Based on the observational study by Dr. Xu and colleagues, which of the following statements about the role of *Haemophilus influenzae* is most likely correct?**

A. The nasopharyngeal environment is likely to be unfavorable to *H. influenzae* colonization

B. At AOM onset, *H. influenzae* is positively associated with *S. pneumoniae*

C. At AOM onset, *H. influenzae* is positively associated with *M. catarrhalis*

D. *H. influenzae* may become a more important cause of AOM in pneumococcal conjugate vaccinated children

**3. Based on the observational study by Dr. Xu and colleagues, which of the following statements about the role of other bacteria in AOM affecting the patient described in question 2 would most likely be correct?**

A. During AOM, *S. pneumoniae* colonization is negatively associated with *S. aureus*

B. Viral-bacterial-host interactions in the nasopharynx during AOM are completely characterized

C. Findings of this study support the role of *S. aureus* as a frequent pathogen of AOM

D. Elimination of *S. pneumoniae* PCV7 strains has resulted in the remaining strains competing less effectively with *H. influenzae* in the nasopharynx and as a cause of AOM infection

## Activity Evaluation

**Table:** 

**1. The activity supported the learning objectives.**				
Strongly Disagree				Strongly Agree
1	2	3	4	5
**2. The material was organized clearly for learning to occur.**				
Strongly Disagree				Strongly Agree
1	2	3	4	5
**3. The content learned from this activity will impact my practice.**				
Strongly Disagree				Strongly Agree
1	2	3	4	5
**4. The activity was presented objectively and free of commercial bias.**				
Strongly Disagree				Strongly Agree
1	2	3	4	5
